# Transient performance prediction of solar dish concentrator integrated with stirling and TEG for small scale irrigation system: A case of Ethiopia

**DOI:** 10.1016/j.heliyon.2022.e10629

**Published:** 2022-09-16

**Authors:** Endeshaw Alemu Bekele, Venkata Ramayya Ancha

**Affiliations:** Faculty of Mechanical Engineering, Jimma Institute of Technology, Jimma University, P.O.Box:378, Jimma, Ethiopia

**Keywords:** Dish concentrator, Stirling engine, Small-scale irrigation, Transient analysis, Thermoelectric generator

## Abstract

The global population growth, climate change effects, and the rapid decline in the stock of fossil fuels increase the demand for food, water, and energy. Irrigation technologies are essential in negating poverty and food insecurity in a developing country while promoting sustainable development goals. However, rain-fed agricultural activities and the lack of modern irrigation technologies in Ethiopia aggravate the problem. This work presents a transient performance investigation of a solar dish concentrator coupled with a Stirling engine and thermoelectric generator for the small-scale irrigation system. A solar dish concentrator with a 2.8 m aperture diameter and 0.4 m depth was used, and Stirling engine analysis was performed using a second-order adiabatic model. System performance was investigated at different operating parameters to predict output power and pump flow rate variation with solar time for a selected irrigation season. Results show that at a heat source temperature of 413.8 K, the thermoelectric unit gives maximum electrical power of 5.2 W at an efficiency of 2.78%. At the same time, the Stirling engine-driven pump provides a cumulative flow rate of 173,594.95 L per day at a thermal efficiency of 18.61%. The output power and pump flow rate reach their maximum at noontime for all selected irrigation seasons. The effect of regenerator effectiveness on the thermal efficiency of the Stirling engine was also examined, and the findings indicate that the Stirling engine's thermal efficiency rises with regenerator effectiveness. Using a solar thermal irrigation system in a location with a high solar radiation potential allows small farmers to generate more income and contribute to the country's food security.

## Highlights

• A pump powered by a Solar Dish Stirling engine was proposed for the irrigation system.
• The transient performance was analyzed using analytical techniques.
• Fluctuations in solar radiation influence the performance of the TEG and Stirling engine.
• The maximum efficiency of the Stirling engine was 18.61%, while that of TEG was 2.78 %.

## 1 Introduction

An estimation shows that by 2050 the global human population will increase by 2.2 billion, global energy demand will be doubled, whereas the demand for food and water will rise by 50% [1, 2]. The growth of populations forces many countries to initiate a Water-Energy-Food nexus policy to meet the demand and reduce environmental impact. Furthermore, the food shortage in developing countries necessitates more farming to alleviate poverty [3]. Lack of improved seeds, hybrid seed multiplication capacity, irrigation technology, and water constraints are significant barriers to Ethiopian agricultural production [4]. In Ethiopia, agriculture is the backbone of the economy, contributing 83.9 percent of exports and 46.3 percent of the country's GDP. However, the contribution of the irrigation system to the country's economy is insignificant [5, 6]. The fast growth of the population underscores the need for sustainable and high-quality access to food.

Around 85% of the Ethiopian population engages in agricultural activities, mainly subsistence, rain-fed crop production, and livestock production [5]. However, millions of smallholder farmers in Ethiopia face food shortages despite significant progress in economic growth. Lack of technology adoption by smallholder farmers often results in the root cause of low agricultural output and food insecurity [6]. Small-scale farms for independent farmers in the country are typically between 0.5 and 2 ha. With the low crop yield and income from the land, they cannot feed their families [7]. Small-scale irrigation enables low-income farmers to produce more crops, generate more revenue, and improve food security [8]. Solar irrigation systems take an advantage over others due to the inconsistency of available fossil fuels and environmental impact issues. Solar thermal technologies are widely used in pumping systems, particularly for irrigation and drinking water requirements [9, 10]. Utilizing hybrid energy as an alternative energy source in agriculture will minimize dependence on fossil fuels and reduce energy demand. Providing cost-effective and appropriate technology in an irrigation system enables farmers to improve crop productivity, livelihood, and food security [11, 12].

Researchers have made numerous attempts to design, model, and evaluate the performance of solar thermal-based water pumping systems utilizing various collectors. Addressing sub-Saharan African irrigation systems, Wazed et al. [3] reviewed solar PV and thermal technology. It is found that the most efficient solar PV for water pumping systems among the various PV technologies is Cadmium Telluride PV solar (CdTe) with a permanent magnet DC motor. The system mainly works during extended durations of sunshine and stores water in a storage tank rather than using a battery. On the other hand, the Stirling pump is the most effective solar thermal irrigation system, operated by a solar concentrator. Additionally, its affordability, simplicity, ease of local manufacture, and environmental friendliness are preferred by smallholder farmers.

Debashis et al. [13] examined the performance of a metal hydride-based solar water pumping system utilizing a parabolic trough. They found that with a 1.5 m^2^ collector area, the system can provide 3000 L per day at a head of 15 m with 1.5% overall efficiency. Moreover, Baral and Kimm [14] investigated the experimental and economic viability of the stand-alone solar ORCs for water pumping systems (for drinking and irrigation) in Nepal using parabolic troughs and evacuated tube collectors. The result shows that at an operating temperature of 120 °C, the system's thermal efficiency is 6%, and the solar ORC prototype has an energy cost of $0.68/kWh.

Mahkamov and Orda [15] used a mathematical model to numerically simulate the internal process and evaluate how well a solar thermal-based water pumping system performed. The outcome demonstrates that the steam proportion of the combined air-steam cycle significantly impacts the system's performance. The findings also imply that when process temperature increases, the output power of the solar thermal water pump increases. On the other hand, performance decreases as condensation loss and water vaporization rise. Kurhe et al. [16] developed a low-temperature heat engine using a diaphragm for water pumping applications. The heat engine operates between 30 and 100 °C using organic working fluid like acetone and n-pentane. At an operating temperature of 85 °C and a head of 5 m, the system can produce 20 L per hour with a 0.25–0.5 % efficiency. Bumataria and Patel [17] reviewed the Stirling engine water pumping system utilizing solar energy as input power. The hot side of the engine attains a heat source temperature between 450–800 °C from solar energy, and the cold side is cooled at a temperature of 35–70 °C. The Stirling engine water pumping system can achieve 52–72 % theoretical thermal efficiency. The system is good hope for a water pumping system in rural areas.

Various researchers have explored employing hybrid solar-TEG to generate power from solar energy [18, 19, 20, 21]. For example, Lertsatitthankorn et al. [18] analyzed the performance of TEG using a parabolic dish with a receiver plate through the different air-cooling systems. Muthu et al. [19] also experimentally analyzed the performance of solar dish concentrators coupled with TEG. A dish concentrator has an aperture diameter of 3.56m, and the receiver is embedded with an absorber plate in the bismuth telluride TEG module. The results show that at a beam solar radiation of 1050 W/m^2^, the maximum receiver temperature was 383 K, the maximum TEG output power was 3.7 W, and the overall system efficiency was 1.68%. With an acrylic cover, the receiver temperature, output power, and efficiency are improved by 1.56%, 2.1%, and 2.51%, respectively.

Bamroongkhan et al. [20] experimentally investigated the performance of a hybrid solar dish photovoltaic-thermoelectric generator. The result indicates that the conversion efficiency of thermoelectric generators and solar PV are 2.96% and 16.69%, respectively. Furthermore, at a temperature gradient of 113.6 °C, the output power obtained from TEG and PV are 2.94 W and 1.93 W, respectively. Çolak and Kıllış [21] developed a photo-heat voltaic thermoelectric generator (PHVT) for the application of irrigation systems. The PHVT module was composed of a sandwich of PV cells with several TEG units connected to the back. TEG module collects the waste heat generated by overheated PV cells. They examined the vertical sandwich and side-by-side two-hybrid configuration possibilities for PV and TEG. The result shows that a vertical sandwiched hybrid system generated 31% more power than a stand-alone PV solar system. Furthermore, if the conditions are kept the same, the side-by-side configuration boosts the output power by 24% more than the vertical sandwiches.

There are also works reported on the performance of the dish concentrator-based Stirling engine for electric power generation. Reddy et al. [22] carried out the performance assessment and energy and exergy analysis of a 50MW parabolic dish Stirling solar power plant. Variations in the solar Stirling engine power plant's efficiency during part-load are taken into account for a year-round performance evaluation. The results show that energy efficiency ranges from 16.83 to 29.18 %, and exergy efficiency varies from 15.57 to 27.09 % for the entire year. Compared to a parabolic dish and Stirling engine, high exergetic losses are found with the concentrator receiver. Lai et al. [23] analyzed the effect of DNI and wind speed on the performance of the dish solar Stirling engine system using a modified theoretical thermally irreversible model. The result shows that solar flux intensity increases the receiver temperature, which improves the heat transferred into the Stirling engine and its thermal performance. At the same time, the wind speed deteriorates the system's performance. With the optimized charged gas mass and more efficient heat exchangers, the system provides an output power of 25kW and overall thermal efficiency of 44%.

Shaikh et al. [24] examined the efficiency and economic assessment of the solar dish-Stirling engine using the System Advisor Model (SAM) tool. A 25kW stand-alone parabolic solar dish/Stirling system's techno-environmental-economic evaluation was carried out. As a result, the system generates 38.6 MW h per year with a net efficiency of 23.39%, achieves a Levelized energy cost of 0.13$/kWh, and emits 762 kg of carbon dioxide annually. In addition, Lashari et al. [25] presented a simulation and mathematical model to carry out the assessment and efficiency improvement of the PSDS system using SAM. The Optical parameters determined by the Opt-geometric model are used in simulation to get the maximum output power.

Various works have also been done by combining the Stirling engine with a thermoelectric generator, utilizing different collectors for power generation and waste heat management. Feng et al. [26] numerically investigated the system consisting of LFR, Stirling engine, and TEG integrated with a PHCA energy storage system. Their work considered the estimation of output power, power generation and storage system efficiency, and investigation of system performance under operating conditions. The result shows that the power generation subsystem could generate 1.46 kW of electricity with an efficiency of 39.52% and an energy storage efficiency of 63.75%. Numerical analysis by Mehrpooya et al. [27] examined the effectiveness of a novel combined energy system comprised of a PDC, Stirling engine, and TEG under various conditions. The result revealed that the optimum total power output and overall efficiencies were 26.2 kW and 39.17%, respectively. Finally, the performance of the dish Stirling engine was theoretically explored by Mohammadnia et al. [28] using a thermoelectric generator as a cavity temperature controller. The cavity's temperature rises around midday due to an increase in DNI and raising the Stirling engine's hot side temperature. Therefore, higher power is generated when the TEG regulates the cavity temperature. It was found that TEG harvested up to 1.2 kW at midday using a dish concentrator with an aperture diameter of 9m. The total power produced by dish Stirling throughout the beginning and ending was increased by 20–30%.

Based on the earlier review, solar dish concentrators can be used with various power generation systems like Stirling engines, photovoltaic cells, steam generators, and thermoelectric generators. The previous literature described the work conducted by employing a solar dish concentrator integrated with TEG and a solar dish Stirling for various applications. Most of the work has focused on evaluating the performance of Dish Stirling engines for power generation applications. However, there was no transient performance evaluation of a solar thermal Stirling engine based on a dish concentrator and a variation of pump output with solar time. This work examines the transient performance of a solar dish concentrator combined with a Stirling and thermoelectric generator for a small-scale irrigation system. For irrigation systems, in particular, it is imperative to study the performance of solar-based Stirling engines and use the waste heat from the dish concentrator receiver to improve overall performance. The evaluation of solar irradiance, performance of the dish concentrator under various operating conditions, variation of TEG output power and efficiency with solar time, the impact of regenerator efficiency on Stirling engine performance, and variation of pump discharge with solar irradiance have all been performed in the present work.

## 2 Materials and methods

### 2.1 Description of the study area

**Figure 1:**
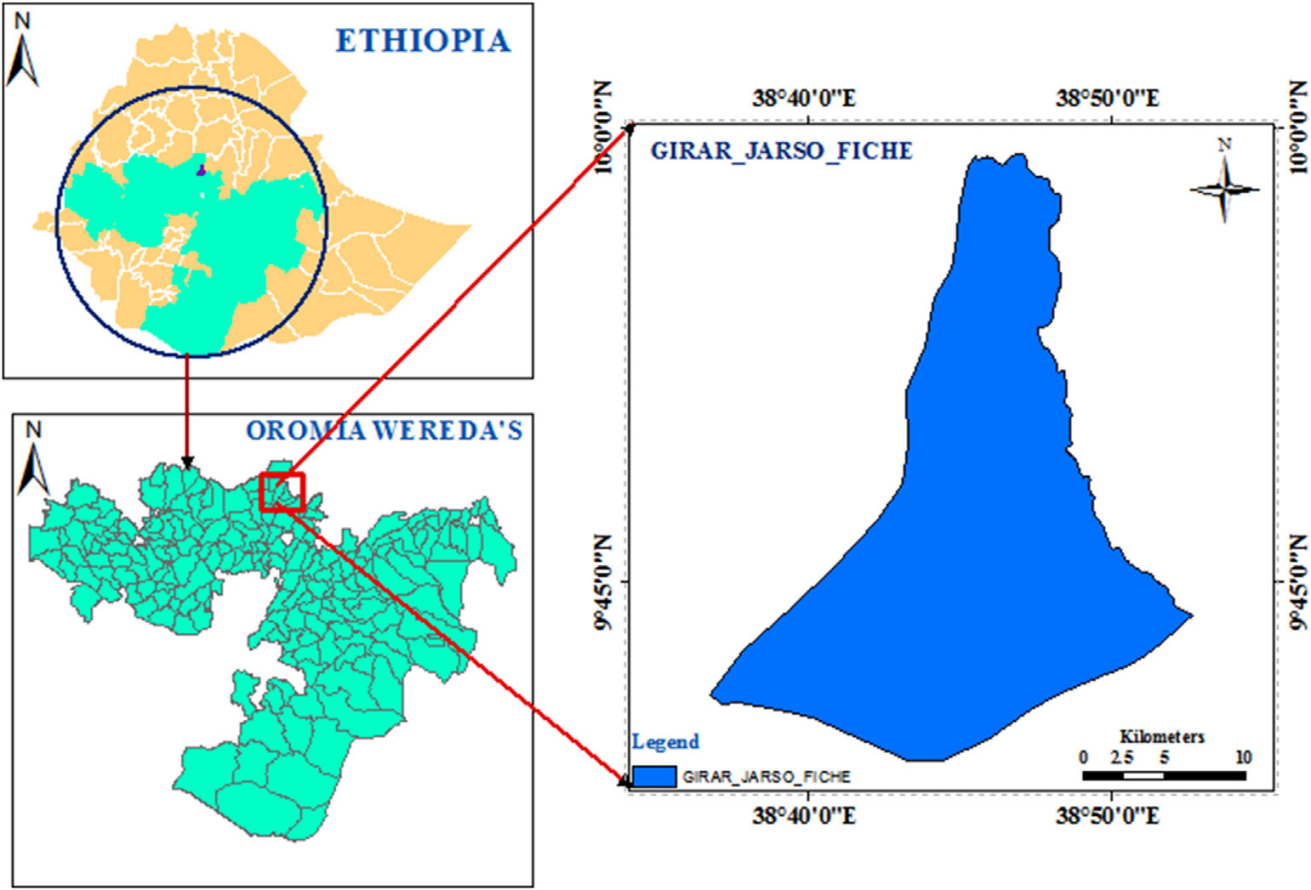
Map of the study area.

The study is carried out in Fiche, north of Ethiopia, located 114 km from the capital city Addis Ababa. It's located at a latitude of 9°48′N and a longitude of 38°44′E, with an elevation between 2,738 and 2,782 m above sea level. The study area's map can be seen in (Figure 1).

### 2.2 Estimation of solar radiation data

**Figure 2:**
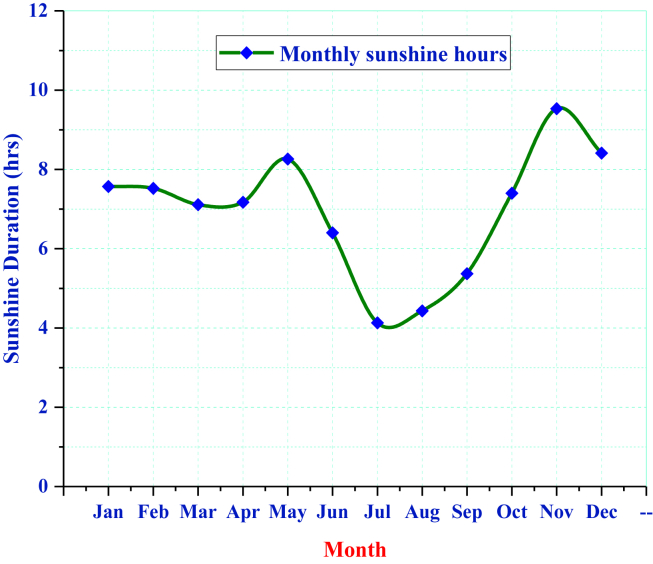
Monthly average daily sunshine hour.

Sunshine hour is an important parameter that helps estimate solar radiation at a given location. NewLoc-Clim is a freeware tool that calculates the local climatic conditions at a given site without observation. It estimates temperature, precipitation, potential evapotranspiration, wind speed, water vapor pressure, and sunshine fraction or hour. The sunshine data are taken from NewLoc-Clim estimator software to estimate solar radiation for a given latitude and longitude (Figure 2). shows that the sunshine hour of Fiche increases from August to November and slightly decreases in the rainy season, where it is low in July with 4.13 h. The result depicts the maximum sunshine hour recorded in November with 9.53 h.

Using the Angstrom-Prescott linear regression model, each month's average diurnal global radiation on a horizontal surface is computed using Eq. (1) [29].

(1) $$\frac{\bar{H}}{\bar{H_{0}}}=a+b\frac{{\bar{n}}_{s}}{\bar{N_{s}}}$$

where; $\bar{H}$ is a monthly average daily global radiation on the horizontal surface, $\bar{H_{0}}$ is monthly average daily extraterrestrial radiation on a horizontal surface, ${\bar{n}}_{s}$ is a monthly average daily hour of sunshine duration in a clear sky, $\bar{N_{s}}$ is a monthly average of the maximum possible daily hour of bright sunshine. The regression parameters “a” and “b” are given as;

$$a=-0.110+0.235cos\varphi +0.323\left(\frac{{\bar{n}}_{s}}{\bar{N_{s}}}\right),$$

$$b=1.449-0.533cos\varphi -0.694\left(\frac{{\bar{n}}_{s}}{\bar{N_{s}}}\right)$$

Where; φ is a local latitude.

The monthly average radiation outside of the atmosphere ($\bar{H_{0}}$) can be obtained using the Klein relationship as given in Eq. (2) [30].

$$\bar{{\text{H}}_{0}}=\left[\frac{24\text{∗}3600}{\text{π}}{\text{I}}_{\text{sc}}\right]\text{∗}\left[\left(1+0.033\text{cos ​}\left(\frac{360\text{n}}{365}\right)\right)\right]\text{∗}$$

(2) $$\left[\text{cos}\varphi \;\text{cosδ}\;\text{sin}{\text{ω}}_{\text{s}}+\frac{\text{π}}{180}{\text{ω}}_{\text{s}}\text{sin}\varphi \;\text{sinδ}\right]$$

Where; n = is the number of days in a year, $I_{SC}$ is a solar constant $\left(1367\frac{\text{W}}{{\text{m}}^{2}}\right),$ δ is a declination angle, and ${\text{ω}}_{\text{s}}$ is a sunset hour angle.

Based on the monthly average daily global radiation on a horizontal surface and the number of bright sunlight hours, the monthly average daily diffuse radiation on a horizontal surface can be estimated through Eq. (3) [29, 31, 32].

(3) $$\frac{\bar{H_{d}}}{\bar{H}}=0.931-0.814\left(\frac{{\bar{n}}_{s}}{\bar{N_{s}}}\right)$$

From the data of monthly average daily global radiation on a horizontal surface, the monthly average hourly global solar radiation on a horizontal surface may be determined via Eq. (4) [29, 31, 32].

(4) $$\frac{\bar{I}}{\bar{H}}=\frac{\pi }{24}\left(a+bcos\bar{\omega }\right)\left[\frac{cos\bar{\omega }-cos{\bar{\omega }}_{s}}{sin{\bar{\omega }}_{s}-\frac{\pi }{180}{\bar{\omega }}_{s}cos{\bar{\omega }}_{s}}\right]$$

$$a=0.409+0.5016sin\left({\bar{\omega }}_{s}-60\right)$$

$$b=0.6609-0.4767sin\left({\bar{\omega }}_{s}-60\right)$$

The monthly average hourly diffuse radiation on a horizontal surface can be computed by Eq. (5). It depends on the monthly average daily diffuse radiation value on a horizontal surface [29].

(5) $$\frac{{\bar{I}}_{d}}{{\bar{H}}_{d}}=\frac{\pi }{24}\left[\frac{cos\bar{\omega }-cos{\bar{\omega }}_{s}}{sin{\bar{\omega }}_{s}-\frac{\pi }{180}{\bar{\omega }}_{s}cos{\bar{\omega }}_{s}}\right]$$

#### 2.2.1 Monthly average daily precipitation and evapotranspiration

**Figure 3:**
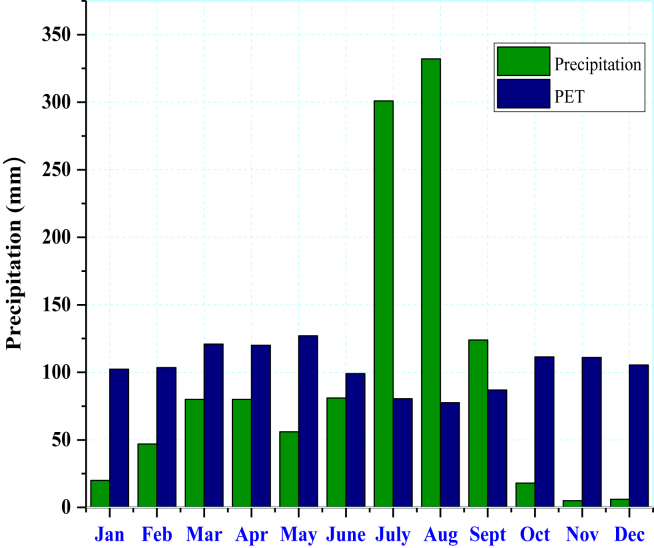
Variation of rainfall and potential evapotranspiration (PET).

**Figure 4:**
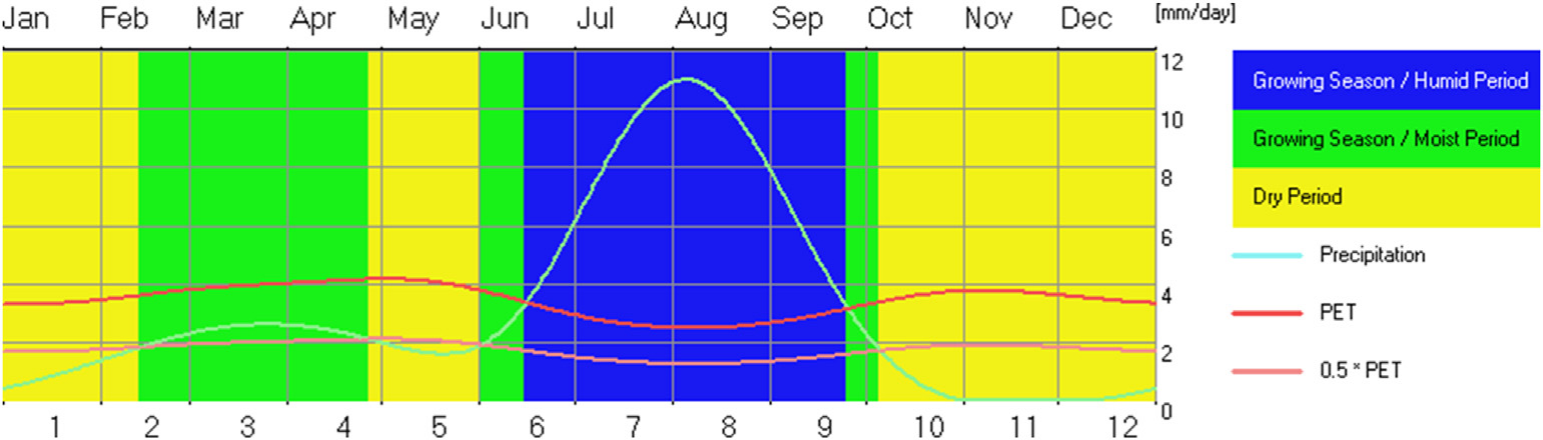
Local growing season relative to potential evapotranspiration and precipitation.

The monthly precipitation and evapotranspiration of the site are obtained from the NewLoc-Clim estimator software. The amount of water needed by crops in the present and future conditions is estimated based on actual precipitation and evapotranspiration. The irrigation system is significant only if the potential evapotranspiration surpasses the substantial rainfall in a site [33]. During this time, water pumping is required for the irrigation systems unless there is no need for water pumping due to excessive rainfall. As shown in (Figures 3 and 4), an irrigation system is only suitable for a specific season (i.e., from October to the end of May). The bar chart in (Figure 3) shows a significant rise in rainfall from 5 mm in November to a maximum of 332 mm in August. Depending on the amount of precipitation and evapotranspiration, the NewLoc-Clim estimator software determines the optimal season for irrigation systems. As indicated in (Figure 4) the growing seasons are classified into humid, moist, and dry periods. Six months are identified as irrigation season based on a growing season and solar radiation data.

## 3 Analyzing the performance of solar dish concentrator coupled with stirling and TEG

### 3.1 Description of the proposed system

**Figure 5:**
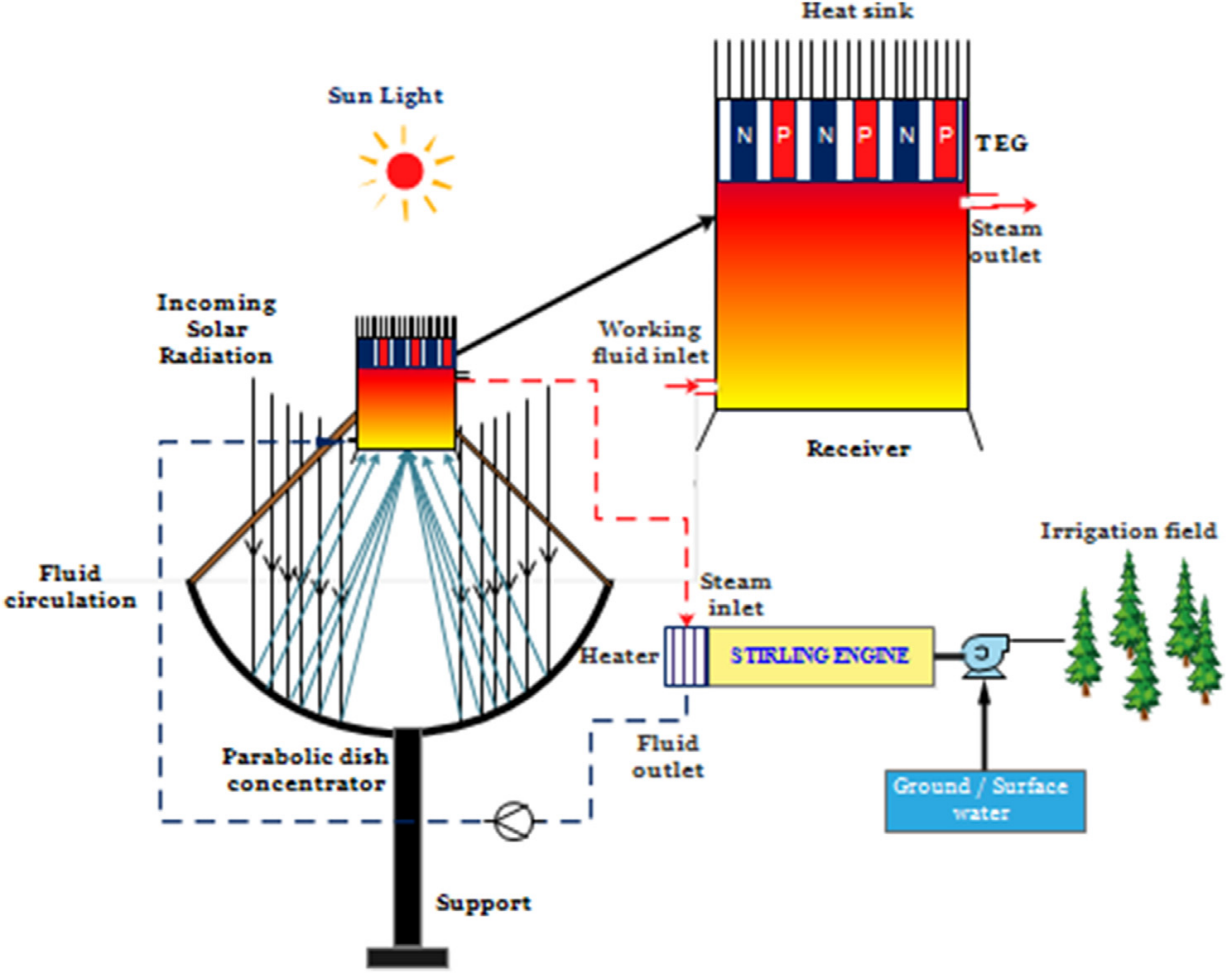
Graphical description of the proposed model (Solar dish concentrator coupled with Stirling and TEG).

The proposed system comprises a dish concentrator, receiver, Stirling engine-driven pump, thermoelectric generator, and fluid circulation component, as presented in (Figure 5). The solar radiation reaching the dish concentrator is concentrated on the absorber located at the focal point. Solar energy is directly transferred from the receiver's surface to the working fluid inside the absorber, resulting in steam generation. The useful heat of the fluid is converted into electrical energy by the thermoelectric generator, which is positioned on the receiver's top. Simultaneously, the outlet fluid from the absorber is used as a heat source for the Stirling engine. The thermal energy from the steam is transferred to the compressed air inside the closed chamber to produce mechanical work utilizing the expansion/compression cycles of the working fluid. The mechanical torque drives the centrifugal pump connected to the Stirling engine to pump the water for an irrigation system.

### 3.2 Analysis of dish concentrator

For the analysis, a dish concentrator with a 2.8m aperture diameter and 0.4m depth is considered [34]. The design parameters of the dish concentrator are estimated, and their values are summarized in (Table 1);

**Table 1:** Solar dish concentrator design parameters and estimated values [34, 35, 36, 37, 38, 39, 40, 41, 42, 43].

Parameters	Formula used	Numerical values	Unit
Aperture area	${\text{A}}_{\text{a}}=\frac{\text{π}{\text{D}}_{\text{c}}^{2}}{4}$	6.157	m^2^
Focal length	$\text{f}=\frac{{\text{D}}_{\text{c}}^{2}}{16\text{∗h}}$	1.2	m
Rim angle	$tan\left(\varphi_{rim}\right)=\frac{1}{\left(\frac{d}{8h}\right)-\left(\frac{2h}{d}\right)}$	59.5	Degree
f/d ratio	$\frac{f}{D_{c}}=\frac{1}{4\;\tan\left(\frac{\varphi_{rim}}{2}\right)}$	0.428	-
Minimum possible receiver diameter	$D_{rec}=\frac{D_{c}*sin\left(\theta \right)}{sin\left(\varphi_{r}\right)}$	0.015	m
Receiver diameter	-	0.2	m
Arch length	$\text{S}=\left\{\frac{\text{d}}{2}\sqrt{{\left(\frac{4\text{h}}{\text{d}}\right)}^{2}+1}\right\}+2\text{fln}\left\{\frac{4\text{h}}{\text{d}}+\sqrt{{\left(\frac{4\text{h}}{\text{d}}\right)}^{2}+1}\right\}$	4.067	m
Concentration ratio	$\text{C}=\frac{{\text{A}}_{\text{conc}}}{{\text{A}}_{\text{rec}}}={\left(\frac{D_{c}}{D_{rec}}\right)}^{2}$	196	-
Rim radius	$r_{r}=\frac{2f}{1+\cos\left(\varphi_{r}\right)}$	1.625	m

#### 3.2.1 Solar dish concentrator efficiency

The optimal performance of the dish concentrator is mainly dependent on the rim angle, the diameter of the receiver, and the concentration ratio of the concentrator [36]. The solar dish concentrator performance is calculated based on an energy analysis using the first law of thermodynamics, considering the optical efficiency and thermal losses incorporated within the concentrator. The thermal efficiency of the dish concentrator should be measured as the ratio of useful thermal heat to input energy as given in Eq. (6). Useful heat is the difference between total heat that reaches the focal plane and total heat losses from the absorber [35]. High optical efficiency and minimal heat losses result in the highest efficiency.

(6) $$\eta_{C}=\frac{Q_{U}}{I*A_{a}}$$

Where; $\eta_{C}$ is a concentrator efficiency, $Q_{U}$ is a useful thermal heat, $A_{a}$ is an aperture area, and I is beam solar radiation.

### 3.3 Stirling engine analysis

A Stirling engine works on a closed thermodynamic cycle with an external heat source via a hot and cold end. Stirling engines have many advantages, like being environmentally friendly, free of emissions, and silent operation [17]. The Stirling engine analysis was performed based on the approach employed by Martini [44]. In this work, the analysis is carried out via an ideal adiabatic second-order analysis using a beta-type Stirling engine, air as the working fluid, and the parameters provided in (Table 2) [[44], [45]]. A second-order analysis depends on an adiabatic modified Schmidt analysis [46].

**Table 2:** Design specification of Stirling engine.

Parameters		Value	Unit
Engine type		Beta	-
Working fluid		Air	-
**Displacer**	Diameter	72	mm
	Stroke length	34	mm
	The swept volume of a displacer	138.4	cm^3^
**Power piston**	Diameter	65	mm
	Stroke length	32	mm
	The swept volume of the piston	106.2	cm^3^
**Regenerator**	Diameter	42	mm
	Height	26	mm
	porosity	79	%
	Void volume	36.02	cm^3^
**Heater**	Diameter	22	mm
	Length	225	mm
	Void volume	85.53	cm^3^
**Cooler**	Diameter	22	mm
	Length	55	mm
	Void volume	20.91	cm^3^
**Phase angle**		90	Degree
**Mean pressure**		1	MPa
**Expansion gas temperature**		413.8	K
**Compression gas temperature**		303.15	K
**Engine speed**		450	RPM

**Figure 6:**
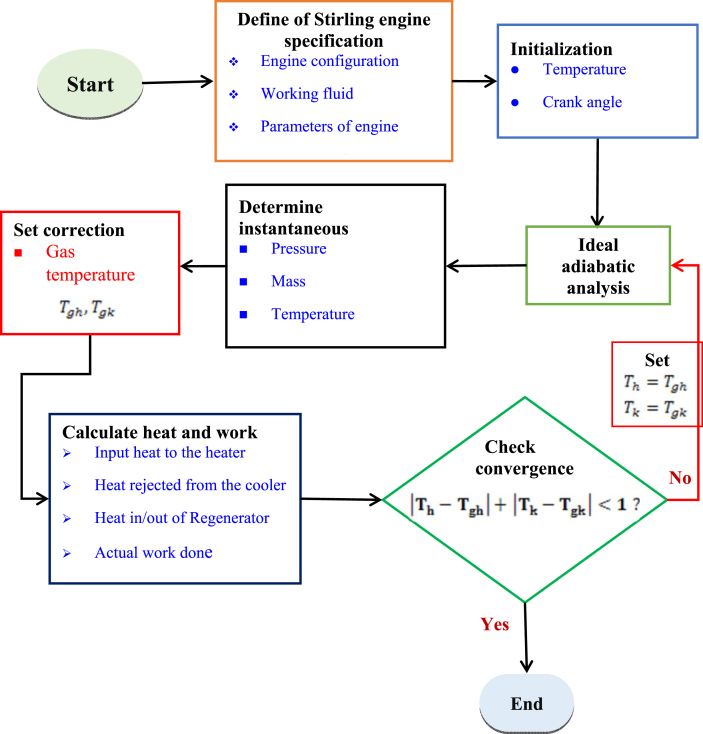
Flow chart of second-order ideal adiabatic analysis.

Without taking into account the heat losses from the system, the second-order adiabatic model is used in this work to analyze the Stirling engine. The numerical analysis flow chart is provided below in (Figure 6). The modified Schmidt analysis considers losses caused by heat transfer and power flow, which requires non-linear time integration of the model equation [47, 48]. ​The investigation was conducted under the following assumptions: The operating fluid temperature within the heat exchanger is constant and equal to the temperature of the exterior wall; the working fluid satisfies the ideal gas law; a perfect regenerator; no loss of friction and leaks [49].

The amount of thermal energy transferred from the dish concentrator to the Stirling engine is estimated by considering the energy balance across the engine [46, 49, 47, 48]. Based on the first law of thermodynamics, the energy equation of the working gas in the engine is computed using Eq. (7) [46].

Rate of heat transfer into cell+Net enthalpy converted in cell=Rate of work done on surrounding+Rate of increase of internal energy in the cell

(7) $$dQ+\left(C_{p}T_{i}m_{i}-C_{p}T_{o}m_{o}\right)=dW_{d}+C_{v}d\left(mT\right)$$

Where; C_p_ is the specific heat capacity of gas at constant pressure, C_v_ is the specific heat capacity of gas at constant volume, $T_{i}$ is the inlet temperature, $T_{o}$ is the outlet temperature, $m_{i}\;$ is an inlet mass flow rate, and $m_{o}\;$ is an outlet mass flow rate.

Heat input to the heater, regenerator, and heat rejected from the cooler heat exchanger is given in Eqs. (8, 9, 10) [46].

(8) $$dQ_{h}=\frac{V_{h}dPC_{v}}{R}-C_{p}\left(T_{rh}m_{rh}-T_{he}m_{he}\right)$$

(9) $$dQ_{r}=\frac{V_{r}dPC_{v}}{R}-C_{p}\left(T_{kr}m_{kr}-T_{rh}m_{rh}\right)$$

(10) $$dQ_{k}=\frac{V_{k}dPC_{v}}{R}-C_{p}\left(T_{ck}m_{ck}-T_{kr}m_{kr}\right)$$

Where $,V_{h}$ $V_{r}$, $V_{k}\;$ are the volume of heater, regenerator and cooler of the Stirling engine heat exchangers, respectively.

The total work done by the Stirling engine is the sum of the work done by expansion and compression of space, as mentioned in Eq. (11) [46].

(11) $$dW=dW_{e}+dW_{c}=P\left(dV_{e}+dV_{c}\right)$$

Eq. (12) shows the network done by the Stirling engine for a non-ideal regenerator [46].

(12) $$W_{NET}=Q_{hi}+Q_{ki}+2*Q_{ri}*\left(1-e\right)$$

Where; $Q_{hi}$ is the heat supplied, $Q_{ri}$ is heat rejected from a cooler, $Q_{ki}$ is regenerator heat during an ideal case, and e is the effectiveness of the regenerator.

The ideal adiabatic thermal efficiency of a Stirling engine is calculated through Eq. (13) [44, 46].

(13) $$\eta_{ideal-Stirling}=\frac{W}{Q_{h}}$$

Where; $Q_{h}$ is the heat supplied to the engine, and W is the network done by the engine.

Eq. (14) indicates the thermal efficiency of the Stirling engine for an imperfect regenerator [46].

(14) $$\eta_{non-ideal}=\frac{\eta_{ideal}}{1+\frac{Q_{ri}}{Q_{hi}}*\left(1-e\right)}$$

The power consumed by the pump is estimated using (Eq. 15),

(15) $$P_{shaft}=\frac{P_{H}}{\eta_{p}}$$

Where; $\;P_{H}$ is a hydraulic power required by the pump, $P_{shaft}$ is the shaft power from the Stirling engine, and $\eta_{p}$ is the efficiency of the pump.

The pump flow rate is estimated based on the pump's hydraulic power and dynamic head via Eq. (16).

(16) $$P_{H}=\frac{\rho gQH_{T}}{3600}$$

Where; ρ is the density of water, g is gravitational acceleration, Q is the flow rate of the pump, and $H_{T}$ is the total dynamic head.

The daily water pumped by the system depends on the pump's capacity and the sunshine hour of the site. It is estimated using Eq. (17).

(17) $$\text{Q}=\frac{\text{Daily water pumped }\left(\frac{{\text{m}}^{3}}{\text{day}}\right)}{\text{Average number of sunshine hour per day}}$$

### 3.4 Performance analysis of TEG

A thermoelectric device is an environmentally friendly and promising solid-state technology for converting thermal energy into electrical energy and vice versa as the temperature gradient occurs between the junctions of the material [50, 51]. This work evaluates the performance of thermoelectric generators using bismuth telluride modules. A summary of thermoelectric module specifications is provided in (Table 3).

**Table 3:** Specification of a commercial thermoelectric module used for analysis [52].

TEHP1-12635-1.2 (Bi_2_Te_3_)		
Parameters	Value	Unit
Hot side temperature	300	^o^ C
Cold side temperature	30	^o^ C
Open circuit voltage	8.3	V
Matched load resistance	2.2	Ω
Matched load output voltage	4.2	V
Matched load output current	1.86	A
Matched load output power	7.8	W
Heat flow across the module	132	W
Heat flow density	10.8	W/cm^−2^

For balancing the total heat flux produced by the TEG module, the rate of heat flow into the hot junction and the rate of heat rejected from the cold junction of the module can be determined using Eqs. (18) and (19) [53, 54].

(18) $$Q_{TEG,h}=N\left(\left(\alpha *I*T_{h}\right)+\left(k*\Delta T\right)-\left(\frac{I^{2}*R}{2}\right)\right)$$

(19) $$Q_{TEG,c}=N\left(\left(\alpha *I*T_{c}\right)+\left(k*\Delta T\right)+\left(\frac{I^{2}*R}{2}\right)\right)$$

Where, $\;Q_{TEG,h}$ is heat rate input on the hot side, $Q_{TEG,c}$ is heat rejected from the cold side, $\;T_{h}$ is the temperature of a hot side, T_c_ is the temperature of the cold side, k is thermal conductivity, N is the number of semiconductor thermocouples, I is the electric current of the module, and R is the resistance of the module.

Energy balance can be used to evaluate the power generated throughout the TEG module [55, 56, 57, 58]. The TEG module's power output was estimated by subtracting the rate of heat input to the hot side of the thermoelectric from heat rejected from the cold side of the thermoelectric, as seen in Eq. (20) [54].

(20) $$P_{TEG}=Q_{TEG,h}-Q_{TEG,c}=N(\left(\alpha *I*\Delta T\right)-\left(I^{2}*R\right))$$

The output electrical power induced by TEG is expressed as

$$P=I^{2}*R_{L}$$

The thermal to electrical energy conversion efficiency of TEG can be calculated through Eq. (21) [54, 57].

(21) $$\eta_{elect,TEG}=\frac{P}{Q_{h}}$$

Where; $Q_{h}$ heat supplied to the hot-side of TEG, P is an output power of TEG and $\eta_{elect,TEG}$ is an electrical efficiency of TEG.

The figure of merit represents how well a thermoelectric generator performs. It is a parameter used to characterize the performance of TEG or measure the amount of thermal energy converted to electrical energy due to temperature gradient [51]. The figure of merit is influenced by material characteristics, including the Seebeck coefficient, thermal conductivity, and electrical resistance. The value of the figure of merit increases with high electrical resistance, low thermal conductivity, and high See-beck coefficient [50, 59]. The maximum energy conversion efficiency of ideal TEG operating under optimal conditions can be estimated using Eq. (22) [50].

(22) $$\eta_{TEG,max}=\left(\frac{T_{h}-T_{c}}{T_{h}}\right)*\frac{\sqrt{1+ZT}-1}{\sqrt{1+ZT}+\left(\frac{T_{c}}{T_{h}}\right)}=\eta_{c}*\frac{\sqrt{1+ZT}-1}{\sqrt{1+ZT}+\left(\frac{T_{c}}{T_{h}}\right)}$$

Where; $T_{h}$ is the hot-side temperature of the module, $T_{c}$ is the cold side temperature, $\eta_{c}$ is Carnot efficiency, and ZT is a figure of merit.

## 4 Result and discussion

### 4.1 The solar energy potential of the site

**Figure 7:**
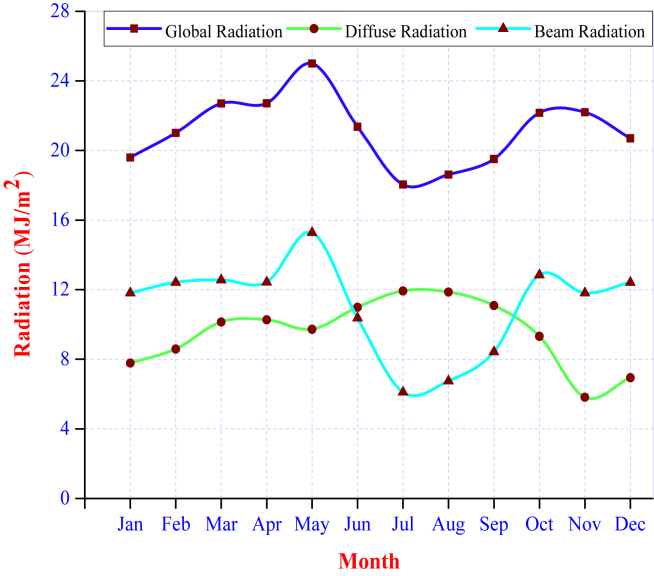
Estimated monthly averages of a daily mean global, beam, and diffuse radiation.

Using the sunshine hour data from the NewLoc-Clim estimator, the monthly average daily global, beam, and diffuse solar radiation on the horizontal surface was calculated through the Angstrom-Prescott correlation as mentioned in Eqs. (1), (2), and (3). The estimated solar radiation value varies from month to month. As seen in (Figure 7), the global solar radiation decreases from May to July and rises from July to November until it reaches its maximum in May with 24.99 MJ/m^2^. Based on the calculated data, it is possible to identify each month with a high potential for solar radiation.

**Figure 8:**
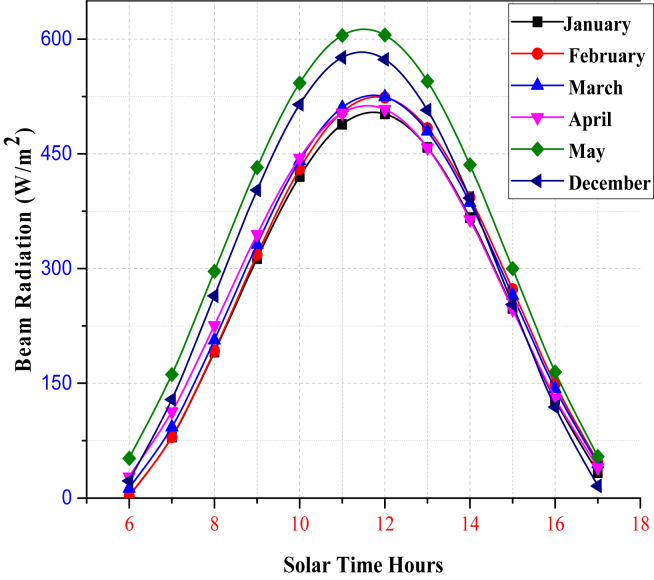
Estimated Monthly Average Hourly Beam Solar Radiation for selected Irrigation Season.

The monthly average daily solar radiation data would be used to compute the monthly average hourly solar radiation. The monthly average hourly beam solar radiation is estimated by subtracting the monthly average hourly global solar radiation from diffused solar radiation. As shown in (Figure 8), the monthly average hourly beam of solar radiation rises from sunrise to a peak at midday before falling till sunset. At noontime, maximum solar radiation reaches 605.34 W/m^2^ in May and a minimum of 502.23 W/m^2^ in January.

**Figure 9:**
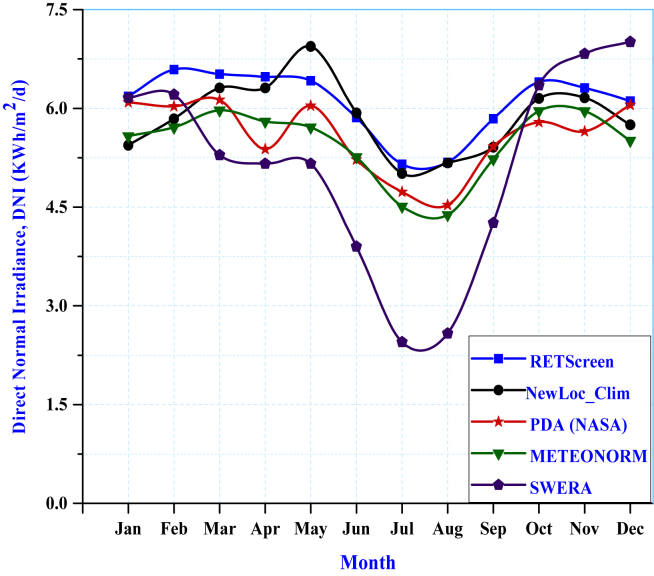
Comparison of estimated monthly direct normal irradiance with solar data taken from different tools.

The estimated solar radiation of the study area is compared with data from SWERA, Meteonorm, RETScreen, and Power data access (PDA). As shown in (Figure 9), the average beam solar radiation at Fiche using NewLoc-Clim is 5.86 kW h/m^2^/day. The average minimum and maximum solar irradiations are 5.11 and 6.08 kW h/m^2^/day for SWERA and RETScreen, respectively. In another case, for NASA surface-based and Meteonorm, it is 5.58 and 5.46 kW h/m^2^/day, respectively. Therefore, the estimated direct solar irradiation by the sunshine hour model is 3.6% lower than that of RETScreen, but 4.77% higher than PDA (NASA).

### 4.2 Performance of solar dish concentrator

**Figure 10:**
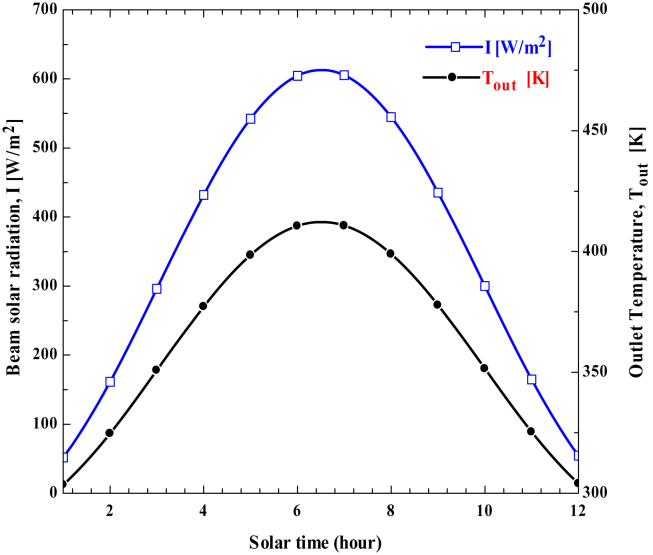
Variation of beam solar radiation and outlet temperature of working fluid with time on May 15.

The performance of concentrated solar power is influenced by direct normal irradiance. Due to variations in solar radiation, the amount of solar energy converted to the thermal heat of working fluid also varies throughout the day. Figure 10 indicates the variation of beam solar radiation and outlet fluid temperature with a time of the day. As seen, beam solar radiation increases from sunrise to a maximum at noontime and then decreases till sunset. The change in outlet fluid temperature depends on the amount of energy transferred to the working fluid and the mass flow rate of the fluid entering the concentrator receiver. Since the outlet fluid temperature is directly related to beam solar radiation, it shows the same trend throughout the day. For the maximum beam solar radiation of 605.3 W/m^2^, the maximum outlet fluid temperature reaches 413.8 K. Higher outlet fluid temperatures result from higher beam solar radiation.

**Figure 11:**
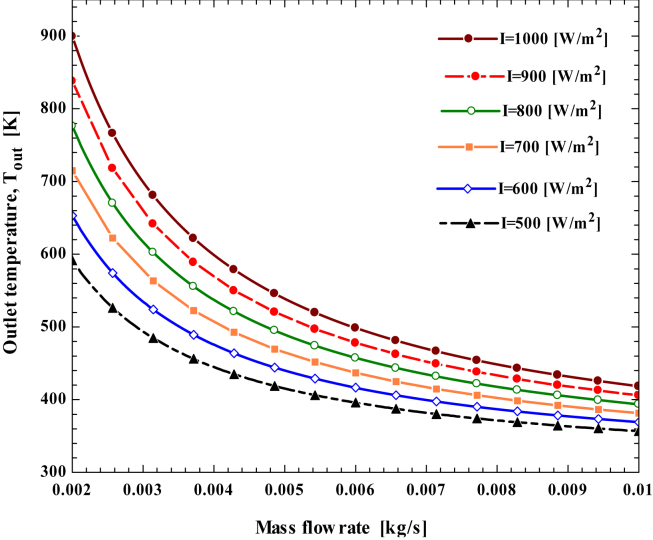
Effect of working fluid mass flow rate on working fluid outlet temperature under different beam solar radiation.

The exit temperature of the working fluid is impacted by the working fluid's thermal heat and mass flow rate. By varying the beam solar radiation from 500 to 1000 W/m^2^ by an interval of 100 W/m^2^, the outlet temperature of the working fluid increases with the beam solar radiation. However, as the mass flow rate rises, the working fluid's exit temperature decreases. As shown in (Figure 11), the outlet temperature is high at the high beam solar radiation and low mass flow rate. For a direct normal irradiance (DNI) of 1000 W/m^2^ and a 0.0065 kg/s mass flow rate, the exit fluid temperature reaches 481.2 K.

**Figure 12:**
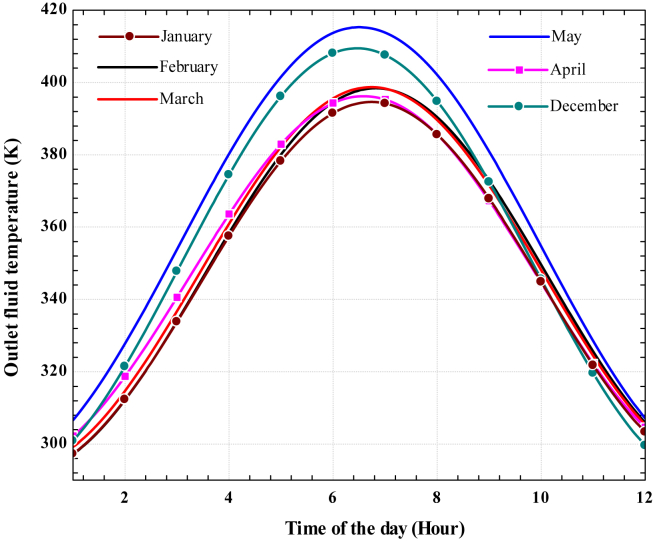
Variation of outlet fluid temperature with time during the day for selected irrigation season.

The fluid exit temperature also changes with solar time due to the variation of beam solar radiation. The variation of outlet working fluid temperature for each irrigation period is shown in (Figure 12). The exit fluid temperature increases from sunrise to a maximum at mid-day and decreases until sunset. The highest and lowest temperatures reached midday in May and January at 413.8 K and 394.2 K, respectively. The maximum outlet working fluid temperature estimated during February, March, April, and December is 398.2 K, 399.3 K, 395.3 K, and 408.1 K, respectively. In general, the outlet working fluid temperature from the receiver of the dish concentrator is directly proportional to the beam solar radiation and inversely proportional to a working fluid mass flow rate. Therefore, the outlet temperature of the working fluid affects the energy that goes to Stirling and the thermoelectric generator.

### 4.3 Variation of TEG performance and output power with time for a given irrigation season

**Figure 13:**
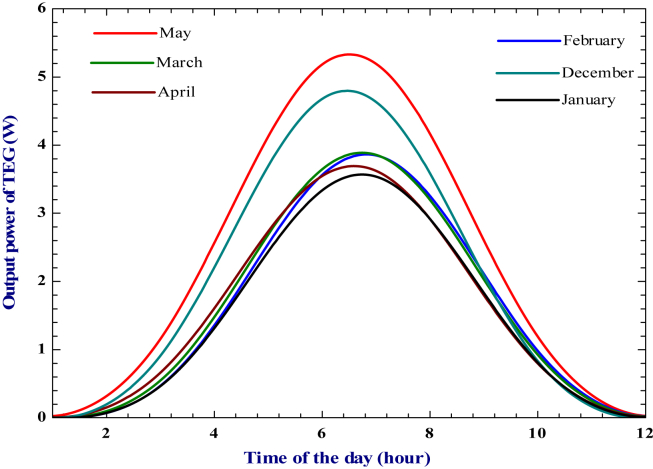
Variation of TEG output power during the daytime for the selected irrigation period.

**Figure 14:**
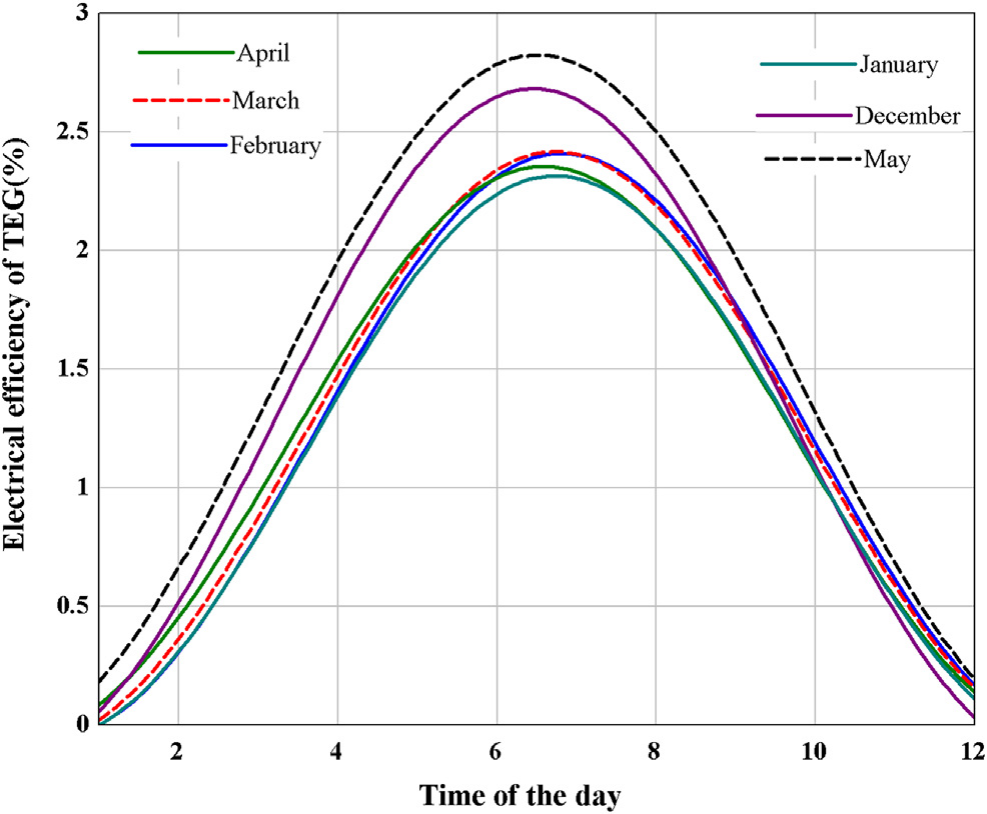
Change of TEG electrical efficiency during daytime for the selected irrigation period.

The performance of a thermoelectric generator is mainly influenced by the construction material and its thermophysical characteristics. The figure of merit and temperature gradient between the hot and cold sides of the module are key factors in TEG's electrical conversion efficiency. The Seebeck coefficient, electrical conductivity, and thermal conductivity are the three main thermoelectric material parameters that affect the figure of merit [59]. Despite material properties, the construction and geometry of the device and the macroscopic heat and electronic transport are essential parameters that have been taken into account in the actual conversion efficiency of TEG [18] (Figures 13 and 14). show that both TEG output power and electrical efficiency are maximum at noontime and minimum during sunrise and sunset. It is seen that higher heat source temperature leads to higher electrical power and efficiency of TEG. For the heat source temperature of 413.8 K, the maximum output and electrical efficiency were found in May, with 5.2 W and 2.78%, respectively.

**Figure 15:**
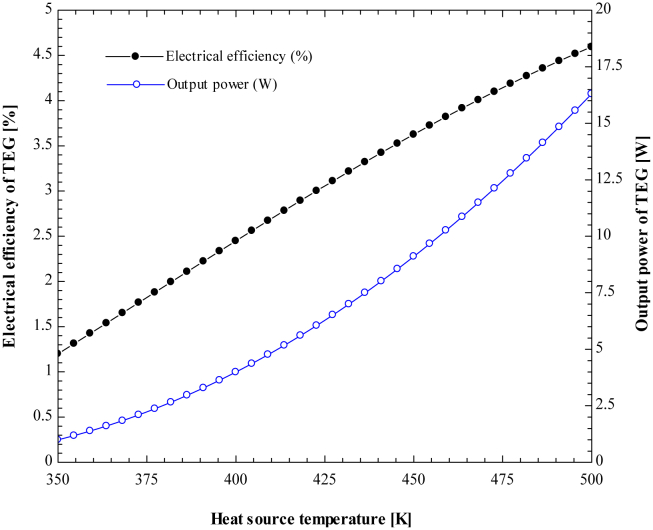
Variation of output power and electrical efficiency of TEG with heat source temperature.

The amount of electrical energy converted by the thermoelectric module depends on the physical properties of the material and the temperature of the heat source. The rate of heat transfer into the modules, Seebeck coefficient, a figure of merit, and electrical resistivity of the material increase as the temperature of the heat source rises. The amount of heat removed from the cold side also depends on the temperature gradient between the hot side and the cold side of the modules (Figure 15). depicts the impact of heat source temperature on TEG's output power and electrical efficiency. By fixing the cold side temperature at 323.15 K, the TEG module's output power and electrical efficiency increase with increasing heat source temperature. So, optimum power output and efficiency are achieved at the maximum hot and low cold-side temperatures.

### 4.4 Validation of thermoelectric electrical efficiency variation with time

The electrical efficiency of the thermoelectric device is influenced by the temperature gradient, physical properties of TEG material (Seebeck coefficient, electrical resistivity, and thermal conductivity), and electrical contact properties (contact resistance and current). Furthermore, the heat source temperature depends on the intensity of solar radiation captured by the concentrator receiver. In contrast, the heat sink temperature relies on the types of cooling systems and the mass flow rate of a cooling medium. The analytical method used to estimate the variation in thermoelectric electrical efficiency over time is compared with experimental findings reported in [20]. The validation was performed considering the same parameters as the literature shown in (Table 4), and it was assumed that the physical and electrical contact properties are identical at all material connections.

**Table 4:** Parameters used for comparison of TEG electrical efficiency [20].

Dish concentrator		Thermoelectric generator	
Diameter (m)	1.5	Material	Bismuth-telluride (TEHP1-12635-1.2)
Focal length (m)	0.57	Cooling type	Forced convection air cooling
Rim angle (degree)	90	Maximum heat source temperature	211.6^o^ C
Depth (m)	0.245	Maximum heat sink temperature	88^o^ C
Maximum beam solar radiation			826 W/m^2^

**Figure 16:**
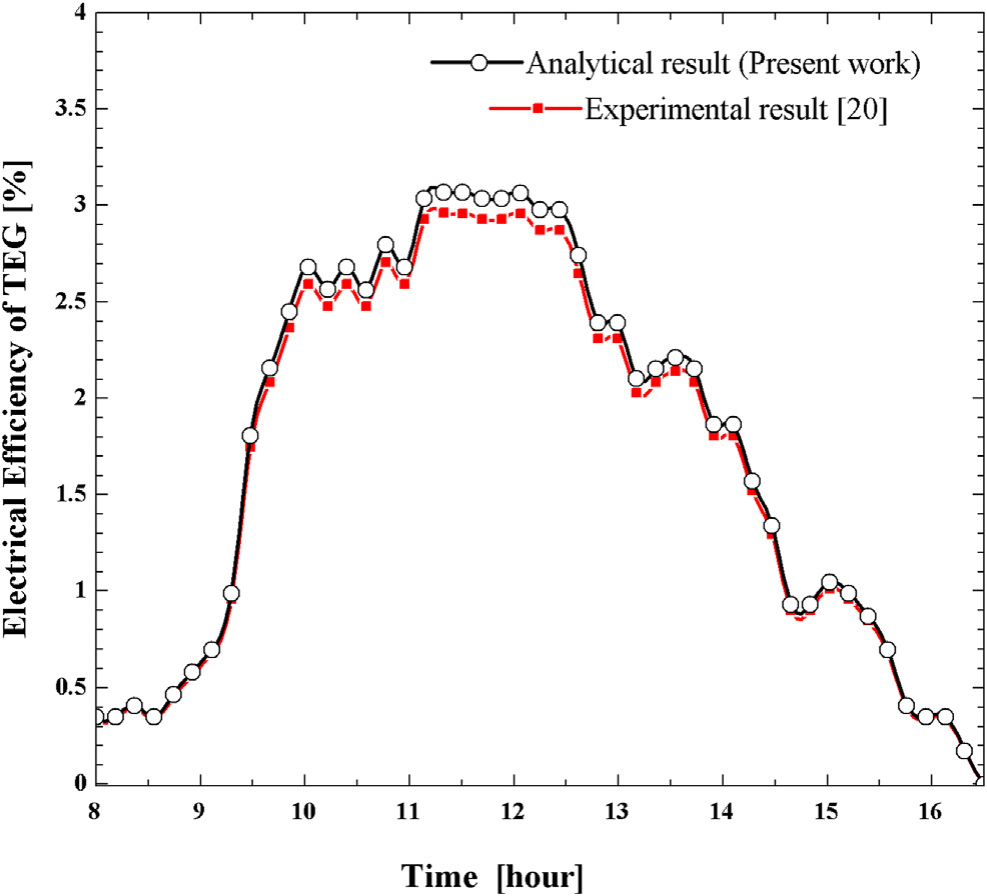
Comparison of the analytically predicted result with experimental for TEG electrical efficiency variation with time.

Lower solar radiation intensity causes a lower heat source temperature, which results in less deviation. But, at noontime, the deviation of calculated results increases due to high solar radiation, which results in a high-temperature gradient, as shown in (Figure 16). Nevertheless, the calculated result agrees very well with the experimental with a maximum deviation of 3.78% at noontime.

### 4.5 Variation of thermal stirling engine performance and output power with time for a given irrigation season

**Figure 17:**
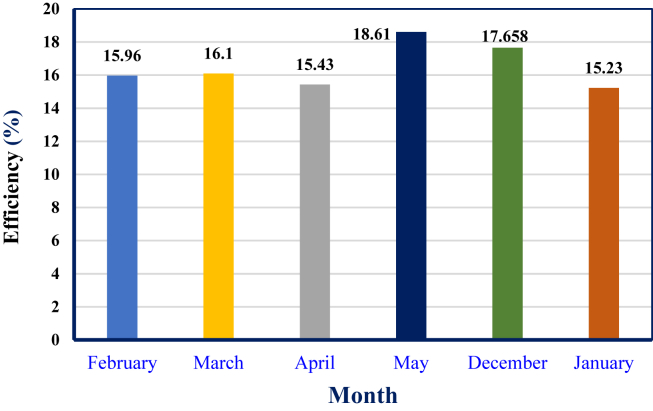
Ideal adiabatic thermal efficiency of the Stirling engine for selected irrigation period.

For each month of the selected irrigation season, the Stirling engine's ideal adiabatic thermal efficiency fluctuates. This is because the heat source temperature affects thermal efficiency, and a higher heat source temperature provides better thermal efficiency. The variance in Stirling engine thermal efficiency for each selected irrigation month is seen in (Figure 17). As shown, thermal efficiency is highest in May at 18.61% and lowest in January at 15.23%.

The pump's output power and flow rate capacity depend on the heat source temperature and solar radiation. The thermal efficiency of the Stirling engine could be increased at high solar radiation due to the increasing heat source temperature for a given cooler heat exchanger temperature.

**Figure 18:**
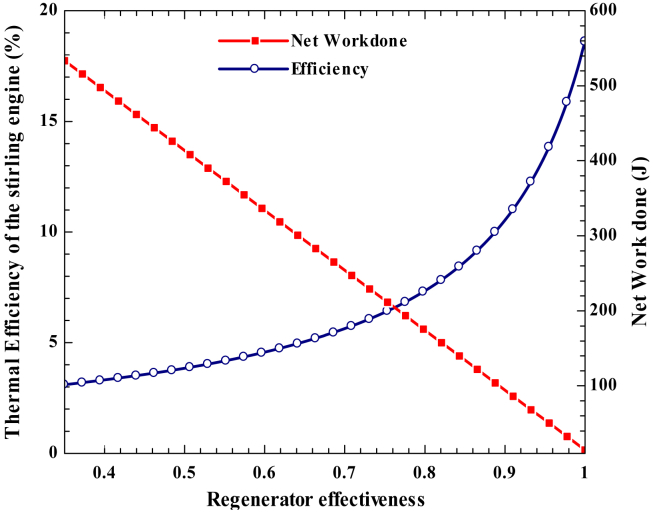
Variation of network done and thermal efficiency of the engine with regenerator effectiveness.

The performance and output power of the Stirling engine are also affected by the regenerator's effectiveness. The net energy of the regenerator during the entire cycle is equal to zero. The heat absorbed by the heater heat exchanger and the work done by the engine decrease in the first half of the process and increase in the second (Figure 18). depicts the impact of regenerator effectiveness on the Stirling engine's network and thermal efficiency. The result shows that when the regenerator effectiveness increases, the Stirling engine's thermal efficiency rises, and the network decreases.

The thermal efficiency of the Stirling engine reaches its maximum for a perfect regenerator. By varying the regenerator's effectiveness from 0.34 to 1, the Stirling efficiency is increased by 15.502%. In the ideal regenerator, the maximum thermal efficiency of the Stirling engine is achieved at 18.61% at a heat source temperature of 413.8 K.

**Figure 19:**
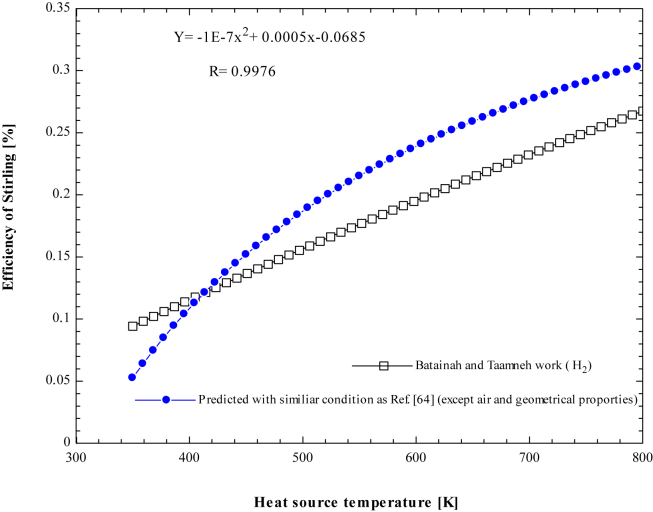
Change of Stirling engine thermal efficiency with a heat source temperature.

The thermal efficiency of the Stirling engine depends on working fluid properties, geometrical and physical characteristics of the engine, heat source temperature, and regenerator effectiveness. The rate of heat transfer into the gas in the expansion space depends on the temperature of the heater. The higher the heat source temperature, the more heat is transferred to gas, increasing the engine's expansion work (Figure 19). shows the effect of heat source temperatures on Stirling engine efficiency for a predicted result considering similar parameters with Batainah and Taamneh [60], except for working fluid and geometrical properties. Initially, air gas pressure is taken to be atmospheric, the cold side temperature is taken as 300 K, and the heater temperature is increased gradually from 350 K up to 800 K.

As can be seen, the thermal efficiency of the Stirling increases with heat source temperature for hydrogen in the work of Batainah and Taamneh [60] as well as for the predicted result using air working fluid. Initially, the efficiency of the Stirling is higher in the case of Hydrogen gas, while it is lower for air at a low-temperature range. At a heat source temperature of 413.8 K, the Stirling engine efficiency is 12.16% for air and 12.12 % for Hydrogen gas. At this point, there is a good agreement between the two situations. Air performs more effectively than hydrogen as the temperature rises above this level. Higher heat source temperature causes thermal losses and induces thermal stress on the material due to increasing gas pressure. Geometrical properties, types of working fluid employed, dead volume, and engine speed causes the deviation between the present work and Batainah and Taamneh [60].

**Figure 20:**
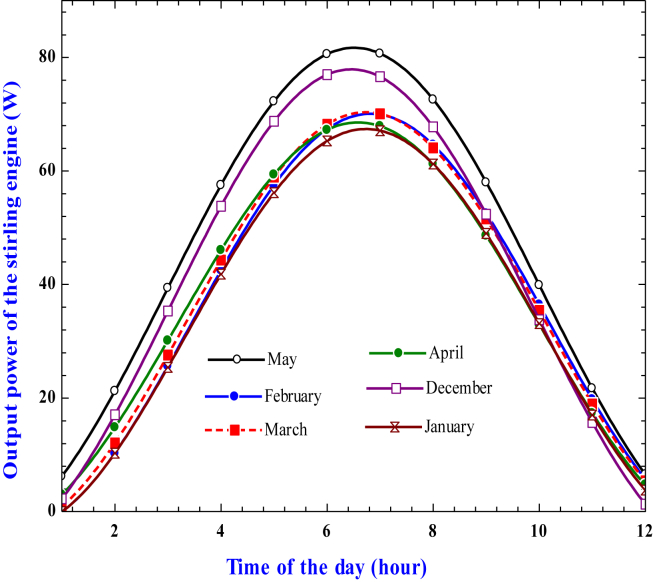
Variation of Stirling engine output power during daytime for a given irrigation season.

The variation of Stirling engine output power with solar time for a selected irrigation period is shown in (Figure 20). On a sunny day, since the heat source temperature varies with the beam of solar radiation, the Stirling engine's output power can be affected indirectly. As can be seen, the output power increases from sunrise to a maximum at mid-day before decreasing again until sunset. High beam solar radiation results in a high heat source temperature so that the high output power of the Stirling engine can be found. The result indicates that the output power is maximum at noontime with 80.68 W in May, 76.6 W in December, 70.6 W in March, 69.94 W in February, 67.9 W in April, and 67.1 W in January.

**Figure 21:**
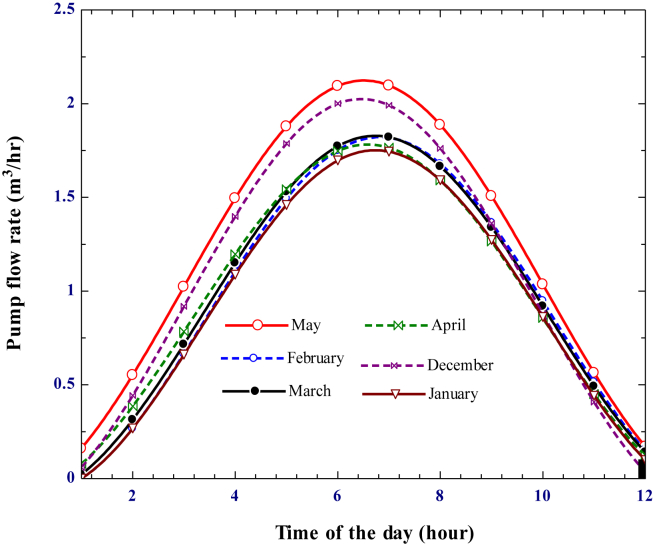
Variation of pump flow rate with time for a given irrigation season.

At a given total dynamic head, the pump's flow rate is influenced by the shaft power from the Stirling engine. The output power and flow rate of the pump have a linear relationship, as shown in (Figure 21). Like power output, the pump's flow rate increases from sunrise to a maximum at noontime before decreasing again until sunset. The flow rate reached its maximum in May, and the pumps operate at their maximum capacity to deliver the water to the irrigation field. Using Eq. (15) and considering the engine's shaft power of 80.68 W and the centrifugal pump's efficiency of 85%, the hydraulic power required by the pump to deliver the water at the specific location should be 68.578 W. The pump flow rate should be estimated based on Eq. (16) for a given hydraulic pump and the dynamic head of the pump. Considering the pump's friction loss and suction head, for a dynamic head of 12 m, the pump flow rate found is 2.097 m^3^/h.

**Figure 22:**
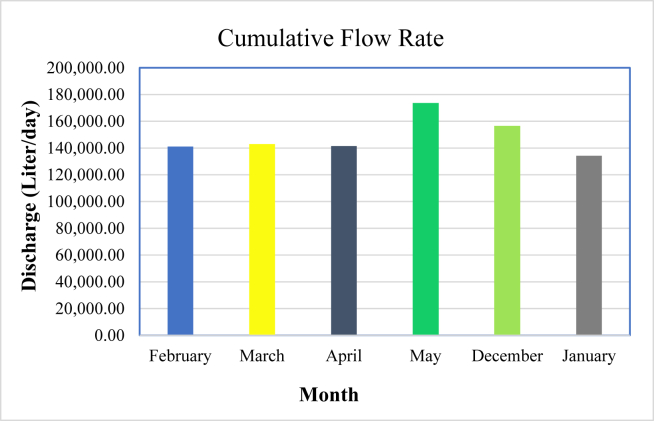
A cumulative flow rate of the pump for the selected irrigation season.

For a given pump efficiency and static head, the pump flow rate depends on the output power generated by a Stirling engine. The pump capacity varies for each selected irrigation period due to the variation in output power of the Stirling engine. At a given head, higher output power results in a higher flow rate (Figure 22). shows the cumulative flow rate for selected average days of each irrigation season. The result indicates that the pump had a minimum capacity of 134,164 L per day in January and a higher capacity of 173,594.95 L per day in May. The cumulative flow rate of the pump in February, March, April, and December is founded to be 141,093.45, 142,665.25, 141,426.89, and 156,494.83 L/day, respectively. Generally, high shaft power from the Stirling engine allows the pump to deliver the required water to the irrigation field at high flow rates.

## 5 Conclusion

The utilization of an effective and affordable solar irrigation system can allow smallholder farmers to generate more income and resolve the risk associated with rainfall, enhancing food security and promoting the attainment of sustainable development goals in a country. This paper presents a transient performance prediction of a solar dish concentrator integrated with Stirling and a thermoelectric generator for small-scale irrigation systems. Solar irradiance was estimated based on the sunshine hour model, and depending on the actual precipitation and evapotranspiration data, six months were selected as the irrigation season. The variation in solar radiation affects the amount of useful thermal energy produced, the dish concentrator's thermal efficiency, the temperature at which Stirling and thermoelectric generators operate, as well as their efficiency and pump flow rates. At beam solar radiation of 605.3 W/m^2^, the dish concentrator's outlet fluid temperature is achieved to be 413.8 K. A thermoelectric generator unit produces 5.2 W of output power at this temperature with an electrical efficiency of 2.78%.

The Stirling engine-driven pump delivers a maximum total capacity of 173,594.95 L per day in May and a minimum of 134,164 L per day in January. In addition, the pump delivers 2,097 m^3^/h with a dynamic head of 12 m and a thermal efficiency of 18.6 %. The thermal efficiency of the Stirling engine increases with heat source temperature and regenerator effectiveness. The thermal efficiency of Stirling rises by 15.502% when the regenerator effectiveness is changed from 0.34 to 1. High beam solar radiation results in a maximum heat source temperature, so the thermoelectric generator's output power and electrical efficiency increase.

Furthermore, the Stirling engine achieves maximum thermal efficiency and high capacity at high direct normal irradiance. The proposed approach demonstrates that the design of a hybrid solar dish concentrator with a Stirling engine-driven pump and thermoelectric generator is effective for small-scale irrigation. Still, further enhancement of TEG performance might be needed by considering different materials and cooling systems as well as optimizing Stirling engine performance while taking system losses into account.

## Declarations

### Author contribution statement

Endeshaw Alemu Bekele: Conceived and designed the experiments, Performed the experiments, Analyzed and interpreted the data; Contributed reagents, materials, analysis tools or data; Wrote the paper.

Venkata Ramayya Ancha: Conceived and designed the experiments, Contributed reagents, materials, analysis tools or data; Wrote the paper.

### Funding statement

This research did not receive any specific grant from funding agencies in the public, commercial, or not-for-profit sectors.

### Data availability statement

Data included in article/supplementary material/referenced in article.

### Declaration of interests statement

The authors declare no conflict of interest.

### Additional information

No additional information is available for this paper.
